# The Gifted Rating Scales - School Form in Greek elementary and middle school learners: a closer insight into their psychometric characteristics

**DOI:** 10.3389/fpsyg.2023.1198119

**Published:** 2023-10-30

**Authors:** Maria Sofologi, Georgia Papantoniou, Theodora Avgita, Anna Dougali, Theodora Foti, Aikaterini-Rafaela Geitona, Aikaterina Lyraki, Anastasia Tzalla, Maria Staikopoulou, Harilaos Zaragas, Georgios Ntritsos, Panagiotis Varsamis, Konstantinos Staikopoulos, Georgios Kougioumtzis, Aphrodite Papantoniou, Despina Moraitou

**Affiliations:** ^1^Laboratory of Psychology, Department of Early Childhood Education, School of Education, University of Ioannina, Ioannina, Greece; ^2^Institute of Humanities and Social Sciences, University Research Centre of Ioannina (U.R.C.I.), Ioannina, Greece; ^3^Department of Early Childhood Education, School of Education, University of Ioannina, Ioannina, Greece; ^4^Laboratory of Neurodegenerative Diseases, Center for Interdisciplinary Research and Innovation (CIRI—AUTH) Balkan Center, Buildings A & B, Aristotle University of Thessaloniki, Thessaloniki, Greece; ^5^Department of Informatics and Telecommunications, School of Informatics and Telecommunications, University of Ioannina, Arta, Greece; ^6^Department of Hygiene and Epidemiology, School of Medicine, University of Ioannina, Ioannina, Greece; ^7^Department of Educational and Social Policy, University of Macedonia, Thessaloniki, Greece; ^8^Department of Computer, Informatics and Telecommunications Engineering, International Hellenic University, Serres, Greece; ^9^Department of Turkish Studies and Modern Asian Studies, Faculty of Economic and Political Sciences, National and Kapodistrian University of Athens, Athens, Greece; ^10^Department of Psychology, School of Health Sciences, University Neapolis Pafos, Pafos, Cyprus; ^11^School of Social Sciences, Hellenic Open University, Patras, Greece; ^12^Laboratory of Psychology, Section of Experimental and Cognitive Psychology, School of Psychology, Aristotle University of Thessaloniki, Thessaloniki, Greece

**Keywords:** Gifted Rating Scales, elementary and middle school children, factorial validity, internal consistency reliability, diagnostic tool

## Abstract

The Gifted Rating Scales - School Form (GRS-S), an evaluation tool for the identification of gifted elementary and middle school children, was the subject of the current study, which focused on its psychometric features (internal consistency reliability and structural validity). Four hundred and eighty-nine teachers (342 women, 139 men, and 8 without gender declaration) used the GRS-S to estimate the dimensions of giftedness in their students for the current study. Particularly, 489 children (253 girls and 236 boys) were evaluated by their teachers. Eight elementary and middle school classes and sixteen 6-month age bands were used to stratify the student population. The scales’ outstanding internal consistency and good factorial validity were revealed by statistical analyses (EFA, CFA, and Cronbach’s coefficients). According to the current research findings, the GRS-S as a reliable and valid assessment tool for identifying gifted students (by their teachers) within the Greek cultural environment.

## 1. Introduction

Recent conceptions of giftedness placed a strong emphasis on the complexity of extraordinary talent. Literature analysis that focuses on the numerous traits of gifted children ranges from broad definitions to more specific ones that combine various elements, including cognitive efficiency, academic ability, leadership, creativity, and even personality traits (Sternberg, 2005). The challenges of accurately identifying talented students are also highlighted in several educational research studies, reflecting the complexity of correct identification and the diversity of this group. A more holistic definition of giftedness that integrates intelligence and non-intellectual skills like self-concept, drive, and creativity is supported by recent research investigations (Park et al., 2007; Johnsen et al., 2010).

High-privileged children can be described as those who, because of their developed cognitive and creative skills, traits, motivations, and preferences, have the ability to complete the curriculum at a significantly faster rate and at a significantly higher level of abstraction and difficulty than many of their peers (Plucker and Callahan, 2014). A comprehensive notion of giftedness that integrates a variety of qualities, features, and skills that can be demonstrated in various ways is now used in research on extraordinarily gifted children (Renzulli, 2011). According to Kaufman et al. (2012), the identification of gifted students has been significantly affected by Marland’s (1972) definition, which was multidimensional in nature. It heralded and illuminated the start of a new era in the study of giftedness because it successfully encouraged the inclination to develop conceptual frameworks for giftedness that emphasize the interaction and synthesis of different traits or characteristics. Additionally, a key component of all new theoretical frameworks is that they view intelligence, which was equated with the idea of giftedness in earlier theories, as a necessary but insufficient condition for children’s remarkable accomplishment (Worrell et al., 2019). Additionally, in recent decades, many academics presented definitions of giftedness that went beyond IQ testing (Acar et al., 2016). Regarding appraising highly talented students, the new, comprehensive models for the identification of giftedness (Jarosewich et al., 2002; Brown et al., 2005; Acar et al., 2016) include criteria and alternative methodologies. The concept of giftedness is viewed as a multifactored ability construct within a network of noncognitive (such as motivation, interests, self-concept, and control expectations) and social moderators that are connected to the giftedness factors (predictors) and the exceptional performance areas (criterion variables). With the use of rating scales, teachers can authentically express their opinions about their pupils across a range of classroom observations and academic activities. Pfeiffer (2012) asserts that gifted children exhibit a higher possibility of making outstanding achievements in one or more fields as compared to other children their age, experience, and opportunity. Furthermore, the literature review reveals promising research data concerning specific scales that assess teachers’ estimations as a screening instrument to help identify gifted students. In an attempt to assess giftedness, rating scales are widely employed as identification methods after IQ testing (Pfeiffer, 2002). The use of teacher rating scales has a long history in the identification of gifted students (Pfeiffer and Blei, 2008). With the use of rating scales, teachers have a real way to condense their opinions on students across a range of academic assignments and classroom observations. Teacher evaluations are important in detecting elements of a person’s talent (such as originality, leadership potential, and intense focus on a particular area) that are difficult to measure using conventional cognitive assessment tools (Pfeiffer, 2015). For the evaluation of gifted students, they might be regarded as a priceless and invaluable resource. Teachers are the first to encounter kids and can spot traits that are strongly related to giftedness, therefore their assessments are crucial (Almeida et al., 2016; Mohamed and Omara, 2020). Researchers stress that by generating useful and concrete observations of their students, teachers can effectively identify them and engage in ongoing interactions with them (Pfeiffer, 2002). Additionally, instructors’ evaluations improve the validity of the assessment process by offering a variety of information about gifted students. This is important because educators’ judgments are crucial because sometimes, due to the remarkable nature of these students’ abilities, standardized IQ tests cannot accurately measure these students’ abilities (Jarosewich et al., 2002; Slater, 2018).

The academic community has developed a few instruments for evaluating gifted children that are geared at instructors and evaluate the students’ traits or actions in several domains as determined by contemporary theoretical models of gifted identification and interpretation (Jarosewich et al., 2002; Renzulli, 2011). One significant rating scale that lays emphasis on giftedness is the Gifted Rating Scale (GRS) (Pfeiffer and Jarosewich, 2003) based on the Munich model for the accurate recognition of giftedness (Heller et al., 2005). Seven reasonably separate ability factor groups—also known as predictors—make up the Munich model. Multiple accomplishments and abilities, as well as different personality traits (motivational, self-perception, control expectation, etc.), as well as environmental influences, or so-called moderators, are the key elements of the Munich model. Thus, the moderators have an impact on how well each individual’s potential (predictors) translates into exceptional performances. This indicates that either academic or extracurricular achievement areas can be linked to giftedness. Furthermore, the family context and school socialization variables are crucial to learning contextual circumstances for fostering outstanding performance across a variety of areas (Heller and Reimann, 2002). This model views giftedness as a multi-factored ability construct that is embedded inside a network of noncognitive (such as motivation, control expectations, and self-concept) and social moderators, as well as performance-related variables. Under the umbrella of this theoretical framework, the GRS’s theoretical underpinning is linked to the multidimensional character of giftedness. Similarly, Pfeiffer and Jarosewich (2003) provided evidence to support the idea that the GRS’s Intellectual Ability factor is connected to the tripartite model’s high intelligence viewpoint, while the factors for artistic talent, academic ability, creativity, and leadership are believed to reflect excellent performance (Pfeiffer, 2012). The GRS, which was created to be accurate and dependable, comprises two forms: a Preschool/Kindergarten Form (GRS-P) for children ages 4.0 to 6.11 and a School Form (GRS-S) for children ages 6.0 to 13.11 (Pfeiffer and Jarosewich, 2003). Six scales (Intellectual, Academic, Creativity, Artistic, Motivational, and Leadership) make up the GRS-S School Form. Each scale has 12 elements (Pfeiffer and Jarosewich, 2003). The overall number of GRS-S items is 72.

The Gifted Rating Scales-School Form (GRS-S) was created using several guiding concepts (Pfeiffer and Jarosewich, 2003). The GRS-S is a rating scale that educators can use to pinpoint the traits of gifted students. When it comes to detecting and evaluating gifted students, the GRS-S offers some advantages. Firstly, administering, scoring, and interpreting the GRS-S is straightforward. However, the validity and reliability of the results are reasonable. IQ testing can also be supplemented with scale scores. Additionally, the scale can be used to evaluate students’ skills over time, enhancing the effectiveness of programs. Finally, tracking students’ development throughout the session can be quite helpful for teachers (Pfeiffer and Jarosewich, 2003). The validity and reliability of the GRS-S scores, for instance, were assessed in Lee and Pfeiffer’s (2006) study using a Korean population. According to the findings of their investigation, coefficient alphas across all scales for teacher ratings were quite high at 0.99. Additionally, the CFA results validated the six-factor solution of the original scale, and Cronbach’s alpha reliability values of the six GRS-S scales for the Arabic version varied from 0.91 to 0.96 (Mohamed and Omara, 2020). Similarly, Li et al. (2008) examined the validity and reliability of GRS-S scores on a sample of Chinese elementary and middle schools and discovered their findings of high reliability were consistent with those reported in the GRS-S test manual (Pfeiffer and Jarosewich, 2003), with alpha values between 0.97 and 0.99. In parallel, Rosado et al. (2015) assessed the validity and reliability of the Spanish-translated Gifted Rating Scales-School Form (GRS) among 618 Puerto Rican school pupils who reside on islands. The Spanish-translated version’s alpha values ranged from 0.98 to 0.99, which is like the values found for standardized samples from the United States (Pfeiffer and Jarosewich, 2003) and a six-factor solution using Confirmatory Factor Analysis (CFA).

## 2. The present study

Greece’s support for gifted education has been insufficient. In the National Educational Curriculum, there is no reference to giftedness that would allow the curriculum to effectively target each student’s learning level in a class that reflects gifted qualities. Since Greece’s educational system does not cater to the demands of its gifted students, there is also no educational identification project on how gifted children can or should be tested and identified in any curriculum or syllabus papers. Furthermore, as no official programs for gifted identification and systematic enrichment programs are available in the Greek educational context the evaluation of gifted students is of vital importance. This study is important for several reasons. First, to our knowledge, there is a small number of studies regarding the adaptation of GRS in Greek culture (Thomaidou et al., 2014; Sofologi et al., 2022), and the adaptation of GRS-P and GRS-S Scales for (Preschool/Kindergarten, Elementary, and Middle School) teachers is still assessed in the European context. Particularly, Sofologi et al. (2022) in their work with kindergarten teachers, indicated the strong factorial and convergent/discriminant validity of the Greek GRS-P measures as well as their excellent internal consistency reliability. The importance of the GRS-P being a valid and trustworthy tool for evaluating gifted students by their educators in the Greek cultural context in relation to the children’s cognitive ability evaluations is therefore emphasized.

However, it remains an unmet need for the adaptation of the GRS-S gifted identification rating scales in Greece, as well. The assessment of the Greek GRS-S psychometric characteristics may help create brief, accurate instruments for identifying, by the educators, the talented and gifted students among Greek elementary and middle school children. It should be noted that the psychometric research, which is presented in the GRS manual (Pfeiffer and Jarosewich, 2003), does not support the two GRS forms’ structural validity. The creators of the Gifted Rating Scales stated that they applied factor analyses to test the internal structure of the GRS, but they did not specify, in the manual (Pfeiffer and Jarosewich, 2003), which kind of factor analyses were conducted. Recently, they have presented these kinds of results from item-level analyses supporting the proposed 6-factor structure as regards mainly the GRS-S (Petscher and Pfeiffer, 2019). Consequently, since no overall score is available, on both GRS-P and GRS-S, we adopted, for testing in our broader research with Greek samples, the proposed (in an item-level analysis) theoretical structure of the GRS-P and the GRS-S consists of 5 and 6 first-order constructs/scales (latent variables), respectively (Margulies and Floyd, 2004; Lee and Pfeiffer, 2006; Benson and Kranzler, 2018; Petscher and Pfeiffer, 2019). To our knowledge, Li et al. (2008) have tested the proposed theoretical structure of the GRS-S consisting of 6 first-order constructs (latent variables), with item-level analyses (CFA), in a Chinese sample, and the indices of their proposed verified model were marginally accepted (RMSEA = 0.077, CFI = 0.91) (Hu and Bentler, 1999; Brown, 2006). Furthermore, the fit indices obtained in the CFA for the Spanish GRS (Rosado et al., 2015) provide moderate support for the six-factor structure (CFI = 0.92, RMSEA = 0.063) (Hu and Bentler, 1999; Brown, 2006). Additionally, although the possibility that a general factor could also account for most of the variance captured by GRS ratings cannot be rejected, the GRS-P and GRS-S scoring model has been examined using a two-factor model approach only by Benson and Kranzler (2018) in the standardization sample in the norming of the GRS-P and GRS-S. According to Benson and Kranzler (2018), the aforementioned possibility is reinforced by the mean scale intercorrelations across age groups for the GRS-P and GRS-S (0.80 and 0.74, respectively), which show high correlations among the scales and indicate a need to investigate whether one or more latent variables (first-order factors) could account for the shared variance among the 5 and 6 proposed scoring structures (as observed variables) of the GRS-P and GRS-S, respectively. In this vein, we conducted a study that aims to shed light on the facets of giftedness, in elementary and middle school education students in Greece, with the Gifted Rating Scales – School Form (GRS-S) (Pfeiffer and Jarosewich, 2003).

The present paper is based on findings concerning the administration of the School Form (GRS-S) in the Greek cultural context and aims to clarify some of the psychometric properties (structural validity, internal consistency reliability) of the GRS-S Greek version. In specific, the objectives of this study were: (a1) the confirmation (at item-level data) of a uni-factorial structure for each of the six scales of the Greek version of the GRS-S, as well as (a2) the test of the six scales (latent variables) correlations, as these correlations depict in confirmatory factor analysis (CFA) (at item-level data) structural model. Considering the finding of Benson and Kranzler (2018) that two general factors (latent variables) have been found (at scale-level data) to account for most of the variance captured by the six GRS-S ratings (measured variables), (a3) another aim of this study was the examination of this possibility in a Greek sample. Finally, the last aim of this study was (b) the evaluation of the internal consistency reliability of each of the six scales of the Greek version of the GRS-S.

## 3. Method

### 3.1. Participants

For the present survey, 489 elementary and middle school teachers participated in the study. More specifically, from the total sample of teachers 342 (69.9%) were women, 139 (28.4%) were men and 8 (1.7%) did not declare their gender. About the time duration, they knew the student whose behavioral and learning characteristics they were going to evaluate, 53 (10.8%) answered they had known their student from 1 to 3 months, 115 (23.5%) answered they knew the student from 4 to 6 months, 133 teachers (27.2%) from 7 to 12 months and 174 teachers (35.6%) more than a year. Regarding how well they believe they know their student, 255 teachers (52.1%) stated they feel they know the child well enough, 176 teachers (36%) stated they feel they know the child very well, while only 43 teachers (8.8%) stated that they do not feel they know it very well. Finally, 3.1% (*N* = 15) did not answer the last two relevant questions. For the GRS-S, 489 students were rated by their teachers. More specifically, 253 were girls (51.7%) and 236 were boys (48.3%). This sample was stratified within the following sixteen 6-month age bands (6:0–6:5, 6:6–6:11, 7:0–7:5, 7:6–7:11, 8:0–8:5, 8:6–8:11, 9:0–9:5, 9:6–9:11, 10:0–10:5, 10:6–10:11, 11:0–11:5, 11:6–11:11, 12:0–12:5, 12:6, 12:11, 13:0–13:5, 13:6–13:11), according to the GRS manual. The demographic variables of students’ age band and gender are displayed in Table 1.

**Table 1:** Group sample of students by age band and gender.

Age band	Group frequency	Percent	Boys	Girls
6:0–6:5	27	5.5%	18	09
6:6–6:11	63	12.9%	26	37
7:0–7:5	33	6.7%	14	19
7:6–7:11	14	2.9%	05	09
8:0–8:5	19	3.9%	12	07
8:6–8:11	17	3.5%	10	07
9:0–9:5	18	3.7%	08	10
9:6–9:11	28	5.7%	15	13
10:0–10:5	33	6.7%	15	18
10:6–10:11	38	7.8%	19	19
11:0–11:5	41	8.4%	22	19
11:6–11:11	60	12.3%	28	32
12:0–12:5	36	7.4%	17	19
12:6–12:11	17	3.5%	09	08
13:0–13:5	27	5.5%	11	16
13:6–13:11	16	3.3%	06	10

### 3.2. Measurements

#### 3.2.1. The Gifted Rating Scale – School Form

The Gifted Rating Scales include a School Form (GRS-S) for ages 6.0–13.11 (Pfeiffer and Jarosewich, 2003). The GRS-S has six scales: Intellectual, Academic, Creative, Artistic, Leadership and Motivation, each with 12 items (for a total of 72 items). Each scale item is rated by the instructor on a nine-point scale with three ranges: 1–3 is seen as below average, 4–6 as average, and 7–9 is regarded as above average. With the help of this rating method, the instructor can determine if a student is below average, average, or above average for each item when compared to other students their age before more precisely grading them on a 3-point scale within the range. The following is a brief description of each of the scales included in GRS-S:

#### 3.2.2. Intellectual ability

This scale measures a student’s perceived verbal and non-verbal intellectual talents according to the teacher. Abstract reasoning (Sternberg, 1985; Zigler and Heller, 2000), problem-solving (Sternberg, 1985, 2000), mental speed (Gagne, 1993), and memory (Sternberg, 1985) are among the aspects of intelligence measured by this scale.

#### 3.2.3. Academic ability

This scale represents how well a teacher thinks a student will handle academic and/or factual topics. Advanced competence and high levels of performance in reading, math, and other disciplines included in the school curriculum, as well as the ease with which one is able to pick up new knowledge and abilities, are all indicators of academic aptitude. Academically gifted students usually possess enormous knowledge bases, including a thorough understanding of their surroundings (Sternberg, 1985, 2000; Schneider, 2000).

#### 3.2.4. Creativity

Using this scale, the teacher assesses each student’s ability to think, act, or generate original, uncommon, or inventive ideas or products. A learner can demonstrate creativity in a number of ways, including how they approach an issue and test out fresh ideas (Cropley, 2000), develop a collaborative project solution, and/or use their imagination. Students who are creative are ingenious, interested, and inquisitive (Sternberg, 1985; Cropley, 2000; Csikszentmihalyi and Wolfe, 2000; Renzulli, 2011).

#### 3.2.5. Artistic talent

Using this scale, the teacher assesses each student’s artistic potential or ability in drama, music, dancing, drawing, painting, sculpture, singing, playing an instrument, and/or acting. Items assess how a student approaches projects, how well they complete assignments, and/or how they use art supplies or creative mediums. Individuals who are artistically brilliant learn artistic abilities more quickly than non-gifted individuals and exhibit more technically sophisticated and mature talents (Bosemer and O’Quinn, 1981; Winner and Martino, 2000).

#### 3.2.6. Leadership ability

The extent to which a student can motivate others to strive toward a common goal is measured by this scale. Items evaluate a student’s conflict-resolution skills as well as their understanding of social dynamics and interpersonal communication.

#### 3.2.7. Motivation

This scale evaluates a student’s tenacity, interest in succeeding, inclination to enjoy challenging activities, and ability to perform effectively under pressure (Ryan and Deci, 2000). Motivation is viewed as the active process that drives and controls a student to achieve, rather than as a trait associated with giftedness. In a variety of contexts, such as while finishing academic or artistic assignments or in control of a group activity, motivation can be observed.

The Gifted Rating Scales – School Form (GRS-S) was translated into the Greek language by Georgia Papantoniou, Chrysoula Thomaidou, and Evangelia Foutsitzi. The International Test Commission (ITC) guidelines (www.intestcom.org) were followed to translate the GRS-S into Greek. To avoid any mistakes and inconsistencies that could disrupt the accuracy of the results, a back translation procedure was also followed (Geisinger, 1994).

### 3.3. Procedure

An attempt was made to select schools and students from different geographic regions. In specific, participants (teachers and students) were recruited from different schools in Greece. Information about the research and its purpose was given to teachers and student’s parents prior to the GRS-S administration. They were also informed of the voluntary nature of the whole procedure and reassured about the confidentiality of all results. All children were attending regular classrooms, without a history of learning difficulties. Teachers were asked to complete individually the ratings based on their observations and not on their inferences. There was no time limit for the completion of the scales and all participants were informed that they were free to withdraw from the evaluation process at any time. Each teacher completed the translated Greek version of the GRS-S once, which is only for one of his/her students. Parents of the students also received a Greek-translated version of the GRS-S and a consent form. Since these are considered personal data, the European Union law that has existed since May 28, 2018, was applied. According to the law, the use of sensitive personal data is allowed only for research reasons. The study’s protocol followed the principles outlined in the Helsinki Declaration and was approved by the Scientific and Ethics Committee of the University of Ioannina (25847/01/06/2021).

## 4. Results

### 4.1. Test of the factor structure of each of the six scales of the GRS-S via exploratory factor analyses application

Initially, to assess the uni-factorial structure of each of the six scales of the GRS - School Form, an exploratory factor analysis was implemented to the data collected from the 12 statements (items) of each nine-point scale, to define the number of its underlying factors. For the extraction of the factors, a principal component analysis with orthogonal Varimax rotation was used, due to the lack of information on the relationships of possible factors since, according to the creators of GRS-S, each subscale seems to have a one-factor structure.

#### 4.1.1. Test of the factor structure of the Intellectual Ability Scale of the GRS-S

The Kaiser-Mayer-Olkin measure was applied to evaluate the total sample suitability, the value of which was Κ.Μ.Ο. = 0.97. Barlett’s sphericity control was statistically significant *χ*^2^ = 7719.45, df = 66, and *p* < 0.001. The analysis revealed one factor with an Eigenvalue >1.0. The Eigenvalue of the factor was 9.82 and the percentage of the explained variance was 81.82%.

#### 4.1.2. Test of the factor structure of the Academic Ability Scale of the GRS-S

The Kaiser-Mayer-Olkin measure was used to evaluate the sample suitability, which was K.M.O. = 0.96. For a further and more concise evaluation of the suitability of the data for factor analysis, Barlett Sphericity Test *χ*^2^ = 7567.71, df = 66, and *p* < 0.001 were applied. The analysis of the data showed one factor with an Eigenvalue >1.0. The Eigenvalue of the factor was 9.55 and the percentage of explained variance was 79.53%.

#### 4.1.3. Test of the factor structure of the Creativity Scale of GRS-S

The Kaiser-Mayer-Olkin measure was used to assess the total sample suitability, the value of which was Κ.Μ.Ο = 0.97. Barlett’s sphericity control was statistically significant *χ*^2^ = 7565.08, df = 66, and *p* < 0.001. The analysis revealed one factor with an Eigenvalue >1.0. The Eigenvalue of the factor was 9.63 and the percentage of the explained variation was 80.24%.

#### 4.1.4. Test of the factor structure of the Artistic Talent Scale of the GRS-S

The Kaiser-Mayer-Olkin measure was used to check the overall sample suitability, which was K.M.O. = 0.97. For a further and more complete examination of the suitability of the data for factor analysis, Barlett Sphericity Test *χ*^2^ = 7702.39, df = 66, and *p* < 0.001 were performed. Data analysis revealed one factor with an Eigenvalue >1.0. The Eigenvalue of the factor was 9.77 and the percentage of explained variation was 81.44%.

#### 4.1.5. Test of the factor structure of the Leadership Ability Scale of the GRS-S

The Kaiser-Mayer-Olkin measure was applied to evaluate the total sample suitability, the value of which was Κ.Μ.Ο. = 0.94. Barlett’s sphericity control was statistically significant *χ*^2^ = 7281.68, df = 66, and *p* < 0.001. The analysis showed two factors with an Eigenvalue >1.0. The Eigenvalue of the first factor was 8.95 and the percentage of the explained variance was 74.61%. The Eigenvalue of the second factor was 1.06 (marginal) with an explained variance of 8.86%. Due to the big difference between the Eigenvalues of the first and the second factor and considering the uni-factorial structure of the scale that has been proposed by its constructors, exploratory factor analysis was used again. For the extraction of the factors this time the criterion of Eigenvalue was replaced by the fixed number of one extracted factor. This factor was found to accept the loadings (>0.80) of all 12 items of the scale and to explain 74.61% of the total variance.

#### 4.1.6. Test of the factor structure of the Motivation Scale of the GRS-S

The Kaiser-Mayer-Olkin measure was used to assess the overall sample suitability, which was K.M.O. = 0.96. For a further and more complete evaluation of the suitability of the data for factor analysis, Barlett Sphericity Test *χ*^2^ = 8091.21, df = 66, and *p* < 0.001 were performed. The analysis of the data revealed one factor with an Eigenvalue >1.0. The Eigenvalue of the factor was 9.72 and the percentage of explained variance was 80.99%.

### 4.2. Test of the factor structure of each of the six scales of the GRS-S via confirmatory factor analyses application

Furthermore, to verify and evaluate the one-factor structure –that has been revealed via EFA– of each of the six scales of the GRS-S for the Greek elementary and middle school teachers’ sample, a set of six confirmatory factor analyses was conducted for the data collected from the 12 items that constitute each of the six 9-point GRS-S scales. Using the Maximum Likelihood estimation approach, each CFA was implemented in the statistical program EQS 6.1 (Bentler, 2005) on a covariance matrix of the 12 items on each nine-point scale. A non-statistical significance of the χ^2^-test indicates that the implied theoretical model significantly reproduces the sample variance–covariance relationships in the matrix (Kline, 2003; Brown, 2006). As this test is sensitive to sample size, model fit was also evaluated by using the root mean squared error of approximation (RMSEA). The RMSEA tests how well the model would fit the population covariance matrix. Specifically, a rule of thumb is that RMSEA ≤0.05 indicates close approximate fit, and values between 0.05 and 0.08 suggest reasonable error of approximation. Models with RMSEA = 0.10 (or RMSEA >0.10) should be rejected (Brown and Cudeck, 1993; Kline, 2003). The Comparative Fit Index (CFI) which is one of the most popular incremental fit indices (Brown, 2006) assesses the relative improvement in the fit of the researcher’s model compared with a baseline model was also used. The CFI indicates a good model fit for values in the range between 0.95 and 1.00, whereas values in the range between 0.90 and 0.95 signify an acceptable fit (Bentler, 1990; see Schweizer, 2010). Additionally, the standardized root mean squared residual (SRMR) was used to evaluate the model fit. The mean absolute correlation residual, or the overall difference between the measured and predicted correlations, is measured by the SRMR. A favorable SRMR value is smaller than 0.08 (Hu and Bentler, 1999; Bentler, 2005; Brown, 2006).

The set of the six CFA models, that were conducted to test the one-factor structure of each of the six GRS-S scales, produced the following indices:

(1) Intellectual Ability Scale, χ^2^ (54, *N* = 476) = 319.99, *ρ* < 0.000, CFI = 0.97, SRMR = 0.02, RMSEA = 0.10 (CI90% 0.09–0.11).
(2) Academic Ability Scale, *χ*^2^ (54, *N* = 481) = 613.07, *ρ* < 0.000, CFI = 0.93, SRMR = 0.03, RMSEA = 0.15 (CI90% 0.14–0.16).
(3) Creativity Scale, *χ*^2^ (54, *N* = 474) = 447.28, *ρ* < 0.000, CFI = 0.95, SRMR = 0.03, RMSEA = 0.12 (CI90% 0.11–0.13).
(4) Artistic Talent Scale, *χ*^2^ (54, *N* = 468) = 554.14, *ρ* < 0.000, CFI = 0.93, SRMR = 0.03, RMSEA = 0.14 (CI90% 0.13–0.15).
(5) Leadership Scale, *χ*^2^ (54, *N* = 482) = 1467.00, *ρ* < 0.000, CFI = 0.81, SRMR = 0.07, RMSEA = 0.23 (CI90% 0.22–0.24).
(6) Motivation Scale, *χ*^2^(54, *N* = 484) = 727.71, *ρ* < 0.000, CFI = 0.92, SRMR = 0.03, RMSEA = 0.16 (CI90% 0.15–0.17) (Hu and Bentler, 1999; Bentler, 2005; Brown, 2006).

Despite the statistical significance of the χ^2^-test, all parameters of the six CFA models were found to be statistically significant (*p* < 0.05) and standardized root-mean-square residual (SRMR) values were below 0.08 indicating also favorable fit for the models tested. The comparative fit index (CFI) values of the five models ranged from 0.92 to 0.97 and were indicative of (marginally) accepted model fit (Kline, 2003; Brown, 2006). Finally, the root mean squared error of approximation (RMSEA) values were above 0.10 indicating a poor fit for all the models tested. These findings of models’ indices, which were acceptable based on CFI and SRMR but poor based on RMSEA values, are consistent with the findings of Petscher and Pfeiffer (2019).

### 4.3. Test of the internal structure of the GRS-S via confirmatory factor analyses application

It should be noted that the aforementioned set of CFA at the item-level data –although they (marginally) verified the proposed by Pfeiffer and Jarosewich (2003) uni-factorial structure for each of the six scales of the Greek version of the GRS-S– were limited as regards the verification (at item-level data) of the GRS-S organization in six underlying factors/latent variables (scales) and the test of the six factors (scales) correlations, as these correlations depict in confirmatory factor analysis (CFA) (at item-level data) structural model. Therefore, we conducted a set of confirmatory factor analyses, using all 12 items from each of the six 9-point GRS-S scales (72 items, total), to verify (at item-level data) the GRS-S organization in the proposed by Pfeiffer and Jarosewich (2003) six-factor structure. One path from every one of the 6 pertinent factors (scales) to each of the 12 items that constitute it, was freed for each confirmatory factor analysis (CFA) model. Cross-loadings were not permitted. CFA was performed twice: At the first performance, latent factors were defined without any covariances between them (Measurement model: Model A). At the second performance, all the latent factors were allowed to freely intercorrelate (Structural model: Model B). For both models, the metric was set by fixing factor variances to 1.0.

Although, all parameters of both the measurement and structural model –that were conducted to test (at the item-level data) the GRS-S organization in six underlying factors/latent variables (scales), as well as the six factors’ correlations–were found to be statistically significant (*p* < 0.05), the indices of the measurement model were not accepted: Measurement model/Model A*, χ*^2^ (2,484, *N* = 436) = 11088.82, *ρ* < 0.000, CFI = 0.82, SRMR = 0.55, RMSEA = 0.089 (CI90% 0.087–0.091).

On the contrary, the structural model was found to fit the data better: Structural model/Model B, *χ*^2^ (2,469, *N* = 436) = 8303.71, *ρ* < 0.000, CFI = 0.88, SRMR = 0.04, RMSEA = 0.074 (CI90% 0.072–0.075). All parameters of Model B were found to be statistically significant (*p* < 0.05) and the standardized root-mean-square residual (SRMR) value was equal to 0.04 indicating a good fit for the model tested. In addition, the comparative fit index (CFI) value was equal to 0.88 indicating a marginally accepted model fit. Finally, the root mean squared error of approximation (RMSEA) value was equal to 0.074 indicating also accepted fit for the structural model (Hu and Bentler, 1999; Kline, 2003; Bentler, 2005; Brown, 2006) that verified the GRS-S organization in the proposed by Pfeiffer and Jarosewich (2003) six-factor structure as well as the six factors’ (scales’) correlations, as these correlations depict in confirmatory factor analysis (CFA) (at item-level data) structural model. The six factors’ (scales’) inter-correlations, as evaluated in the CFA Structural Model (Model B) are depicted in the second columns of Table 2.

**Table 2:** Inter-correlations between the six scales of the Gifted Ratings Scales - School Form.

Scales	Intellectual ability	Academic ability	Creativity	Artistic talent	Leadership ability	Motivation
Intellectual ability	1.000					
Academic ability	0.910** 0.939*	1.000				
Creativity	0.790** 0.817*	0.810** 0.838*	1.000			
Artistic talent	0.663** 0.673*	0.698** 0.711*	0.756** 0.759*	1.000		
Leadership ability	0.742** 0.755*	0.788** 0.803*	0.719** 0.730*	0.683** 0.696*	1.000	
Motivation	0.775** 0.786*	0.849** 0.863*	0.720** 0.736*	0.670** 0.679*	0.811** 0.826*	1.000

At the first column of this table are depicted the six scales’ inter-correlations, as they were calculated with the Pearson correlation coefficient r. The symbol ** is equal to *p* = 0.01 (2-tailed).

At the second column of this table are depicted the six factors’ (scales’) inter-correlations, as they were evaluated in the CFA Structural Model (Model B). The symbol * is equal to *p* = 0.05.

It should be highlighted that the set of CFA (Models A & B) at the item-level data –although they verified the proposed by Pfeiffer and Jarosewich (2003) GRS-S organization in six underlying factors/latent variables (scales) and the six factors’ (scales’) inter-relations, as these correlations depict in confirmatory factor analysis (CFA) (at item-level data) structural model– were limited as regards the identification of the second-order underlying factors’ number in which the GRS-S six first-order factors’ (scales’) could be organized.

Given that the uni-factorial structure of each of the six scales of the Greek version of the GRS-S was confirmed using item-level data (Model B) and taking into consideration the finding by Benson and Kranzler (2018) using scale-level data (rather than item-level data), either (a) a general factor (latent variable) or (b) two general factors (latent variables) have been found (from the execution of EFA, CFA, and ESEM [Exploratory Structural Equation Model], respectively) to account for most of the variance captured by the six GRS-S ratings (measured variables), we followed Benson and Kranzler (2018) methodology. To be more precise, we used both exploratory factor analysis (EFA) and confirmatory factor analysis (CFA) on the scale-level data of all six scales of the Greek GRS-S to determine the number of latent variables (underlying factors) that make up their organization. Scales were first treated as measured (observed) variables in EFA and then in CFAs, which were conducted at the scale-level data, because neither EFA nor CFA were run at the item level but rather at the total scores for the verified factor structure of each scale of the GRS-S: the Intellectual Ability scale, the Academic Ability scale, the Creativity scale, the Artistic Talent scale, the Leadership Ability scale, and the Motivation scale.

To conduct the EFA, we calculated the sample adequacy using the Kaiser-Meyer-Olkin and Bartlett’s Sphericity Tests (K.M.O. = 0.888 and 2 = 2792.871, df = 15, *p* = 0.000, respectively), and we also utilized the scree plot to decide how many variables to keep in the study. Due to Benson and Kranzler (2018) discovery of the one-factor solution, a principal component analysis (PCA) with orthogonal Varimax rotation was used for the factor extraction of the factors. One factor with an eigenvalue larger than 1.00 was produced by the analysis. The first factor’s eigenvalue was 4.81, and 80.12% of the variance was explained by it. All other eigenvalues were far below the minimal retention standard. This finding is consistent with Benson and Kranzler (2018) EFA one-factor solution.

Then, a CFA was applied to confirm the model (Model D1) with a single general factor. The indices of the Model D1 were: *χ*^2^ (9, *N* = 436) = 181.49, *ρ =* 0.000, CFI = 0.94, SRMR = 0.04, RMSEA = 0.21 (CI90% 0.18–0.24). All parameters of Model D1 were found to be statistically significant (*p* < 0.05), the standardized root-mean-square residual (SRMR) value was below 0.05 indicating a good fit for the model tested, and the comparative fit index (CFI) value was found to fell at the highest boundary of the marginal range of 0.90–0.95, and was indicative of accepted model fit (Kline, 2003; Brown, 2006). However, the chi-square goodness-of-fit test was statistically significant resulting in a rejection of the null hypothesis of good fit, and mainly, the root mean squared error of approximation (RMSEA) value was equal to 0.21 indicating a poor fit for the model tested.

Since the aforementioned finding, that the RMSEA value of the single-factor model (Model D1) provides a poor fit to the GRS-S (at scale level) data, was similar to Benson and Kranzler (2018) CFA one-factor solution, we continued with the test of their improved alternative bi-factor solution –which had been found from their conduction of Exploratory Structural Equation Modeling– in order for us to be able to identify the number of the six GRS-S organization’s underlying factors (latent variables) in the Greek cultural context. Then, a CFA was applied to confirm the aforementioned alternative bi-factor model (Model E1), which included a general factor as well as a single group factor reflecting covariance among ratings of the artistic ability scale, leadership ability scale, and motivation scale, that is independent of the general factor and seemingly nonintellectual in nature.

The indices of the measurement Model E1 were: *χ*^2^ (6, *N* = 436) = 101.21, *ρ = 0*.000, CFI = 0.97, SRMR = 0.03, RMSEA = 0.19 (CI90% 0.16–0.22). Although the standardized root-mean-square residual (SRMR) value was below 0.05 and the comparative fit index (CFI) value was equal to 0.97 indicating a good fit for the model tested, the chi-square goodness-of-fit test was statistically significant resulting in a rejection of the null hypothesis of good fit and the root mean squared error of approximation (RMSEA) value was equal to 0.19 indicating poor fit for the model tested. The aforementioned indices of Model E1 were similar to the indices of Benson and Kranzler (2018) alternative bi-factor model. Furthermore, in Model E1 –of our bi-factor solution– the second single group factor reflecting covariance among ratings of artistic ability scale, leadership ability scale, and motivation scale, was marginally found (*p* = 0.12) to not be statistically significant (*p* < 0.05), according to the Wald Test.

As the restrictive measurement Model D1 and Model E1 provided inadequate fit, we proceeded with the identification of the areas of these models that contributed most to the misfit. Residual analysis was conducted, and the Wald Test was performed. Different models were tested and the modifications –covariances (interrelations) between errors of observed variables– indicated by the aforementioned tests were included in the model being tested each time. The necessity of adding these interrelations arose from the fact that these observed variables were differentially prone to social desirability (Brown, 2006). In the final Model D2 was added seven interrelations and in the final Model E2 was added four interrelations. All the modifications, that were added, were statistically significant and improved the fit of the final models D2 and E2 on all indices: Model D2, *χ*^2^ (2, *N* = 436) = 0.744, *ρ =* 0.689, CFI = 1.000, SRMR = 0.003, RMSEA = 0.000 (CI90% 0.000–0.071), and Model E2, *χ*^2^ (2, *N* = 436) = 0.744, *ρ =* 0.689, CFI = 1.000, SRMR = 0.003, RMSEA = 0.000 (CI90% 0.000–0.071).

**Figure 1:**
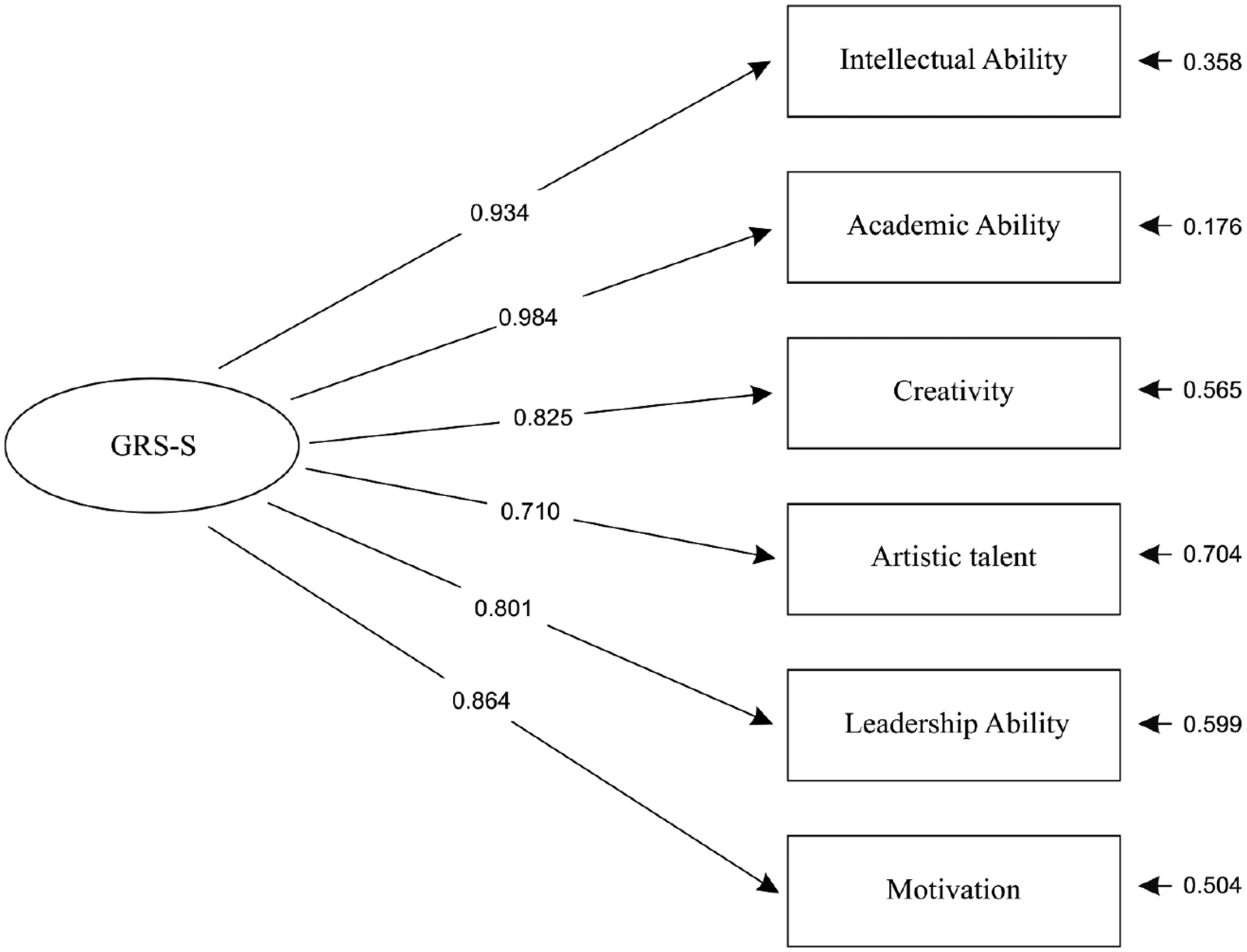
The one-factor CFA model (Model D2) of Gifted Rating Scales – School Form. Note 1. GRS-S: A general factor (latent variable) reflecting common variance in all ratings. Note 2. Covariances (interrelations) between errors of observed variables: Creativity – Intellectual Ability = 0.120, Motivation – Intellectual Ability = -0.165, Artistic Talent – Creativity = 0.425, Leadership – Creativity = 0.166, Leadership – Artistic Talent = 0.268, Motivation – Artistic Talent = 0.151, Motivation – Leadership = 0.389. Note 3. All parameters of Model D2 are statistically significant (*p* < 0.05).

**Figure 2:**
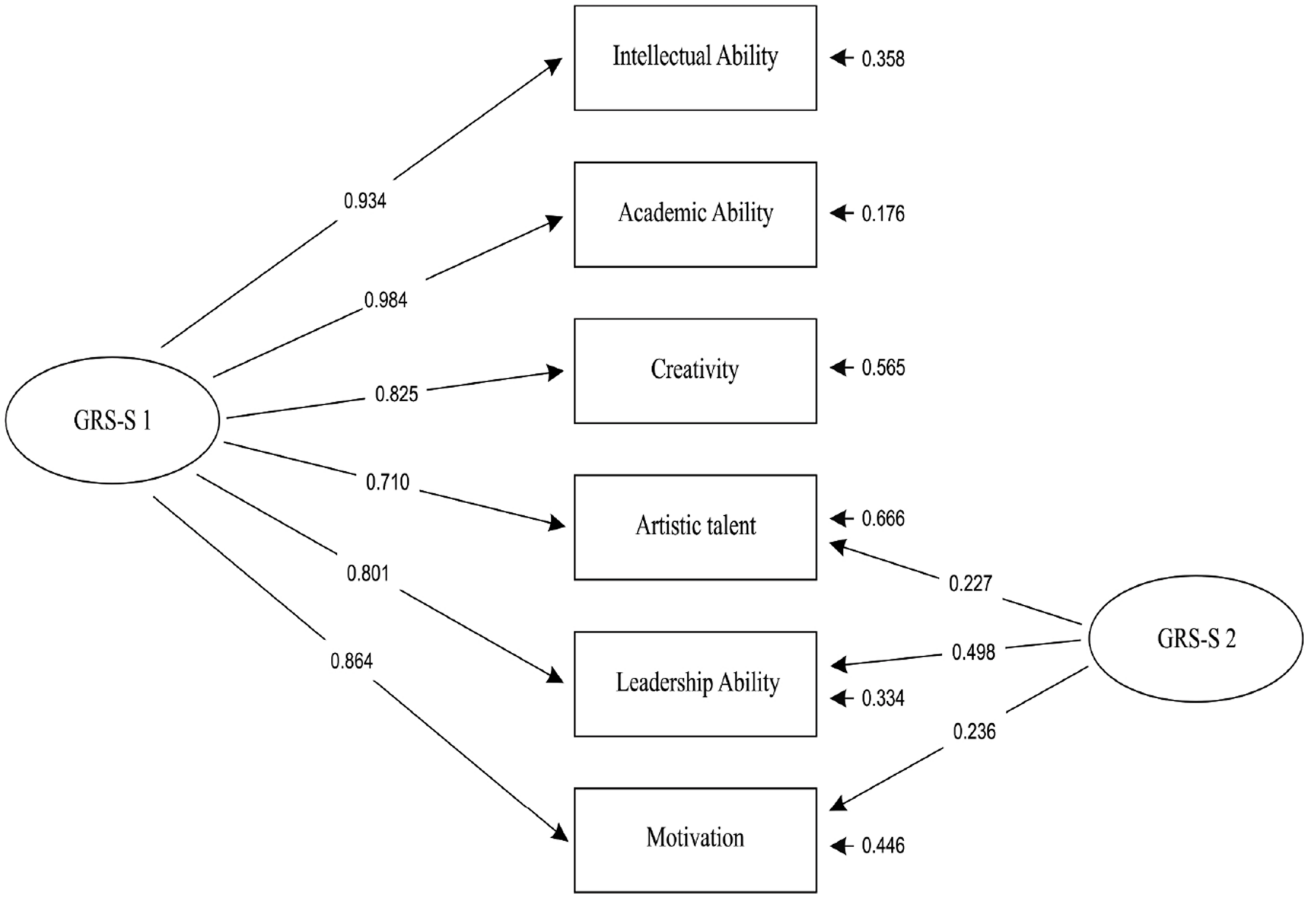
The bi-factor CFA model (Model E2) of Gifted Rating Scales – School Form. Note 1. GRS-S 1: A general factor (latent variable) reflecting common variance in all ratings. GRS-S 2: A group factor (latent variable) reflecting nonintellectual characteristics. Note 2. Covariances (interrelations) between errors of observed variables: Creativity – Intellectual Ability = 0.120, Motivation – Intellectual Ability = -0.187, Artistic Talent – Creativity = 0.449, Leadership – Creativity = 0.297. Note 3. All parameters of Model E2 are statistically significant (*p* < 0.05).

As can be seen, the indices of Model D2 and E2 were identical, although the two models’ parameters, equations, variances, and covariances were slightly different. The identification of the two models’ indices can be explained by taking into account that both models were based on the same number of cases (436), and they had the same number of dependent variables (6), almost the same number of independent variables (7 & 8, respectively), the same number of free parameters (19), and almost the same number of fixed nonzero parameters (7 & 8, respectively). Finally, both models met the criterion that all of their parameters had to be statistically significant (*p* < 0.05) and not to be dropped by the Wald Test. Results revealed that both the one-factor model (Model D2), as well as the alternative two-factor model (Model E2), provided exactly the same excellent fit (Hu and Bentler, 1999; Kline, 2003; Bentler, 2005; Brown, 2006) to the Greek version of GRS-S (at scale level) data and should be interpreted as complementary to each other. This finding is slightly different from Benson and Kranzler’s (2018) proposed bi-factor solution for the US version of GRS-S. R^2^ values for the general factor ranged from 0.50 to 0.97 (Model D2) and from 0.56 to 0.97 (Model E2). Figure 1 (Model D2) and Figure 2 (Model E2) present standardized loadings and residual variances for observed variables.

### 4.4. Test of the inter-correlations of the six scales of the GRS-School Form

Additionally, the inter-correlations of the six scales of the GRS - School Form, were calculated with the Pearson correlation coefficient *r*. As shown in the first columns of Table 2, all correlations between the scales were positive and statistically significant at the level *p* = 0.01. The scales of GRS-S are interrelated to each other to a moderate up to a high degree. Intra-correlations range from 0.67 between the Motivation and Artistic Talent scale to 0.91 between the Academic Ability and Intellectual Ability Scale. The six factors’ (scales’) intercorrelations, as evaluated in the CFA Structural Model (Model B) are also depicted in the second column of Table 2. All of them are statistically significant (*p* < 0.05).

### 4.5. Test of the GRS-S internal consistency reliability

Cronbach’s alpha coefficients were also used to assess the GRS-S’s internal consistency reliability. The Cronbach’s *α* internal consistency coefficients of all scales of the Greek version of the GRS-S, for the corresponding sample of the present study, ranged between 0.968 and 0.980 and were:

(1) Intellectual Ability Scale*, α* = 0.980.
(2) Academic Ability Scale*, α* = 0.976.
(3) Creativity Scale*, α* = 0.977.
(4) Artistic Talent Scale*, α* = 0.979.
(5) Leadership Scale*, α* = 0.968.
(6) Motivation Scale*, α* = 0.978.

The alpha internal consistency coefficients were outstanding for all scales of the GRS-S Greek version. These findings closely match those of Pfeiffer and Jarosewich (2003).

## 5. Discussion

For Greek elementary and middle school teachers and students, the present study assessed the psychometric qualities of each of the six scores from the Gifted Rating Scales. Assessment procedures play a vital role in identifying students with high aptitude levels. The GRS-S has been shown to be a reliable instrument for identifying giftedness under the guise of appropriate or accurate assessment. Through the exploratory analysis of factors, our prediction that the Greek version is one-dimensional and bi-factor was confirmed with reference to the factorial structure of the various scales of the GRS-S. Their one-factor structure is congruent with the GRS-S theoretical construction, which states that they represent several giftedness dimensions as determined by contemporary multidimensional theoretical models of giftedness identification (Pfeiffer and Jarosewich, 2003). According to our factor results, the original measurement model’s evidence surpassed both a unidimensional and a bi-factor model (Petscher and Pfeiffer, 2019). Our results revealed the existence of a sizable general component for the GRS-S; this conclusion is in line with that of Benson and Kranzler (2018). Application of the EFA results supported a single-factor solution for the GRS-S. The use of EFA and CFA revealed the existence of a sizeable general factor, which was found to account for a sizable portion of the GRS-S variation, which is also significant regarding the study’s aims. These findings are consistent with those reported by the original authors (Pfeiffer and Jarosewich, 2003), as well as with revisions made later (Lee and Pfeiffer, 2006; Li et al., 2008; Rosado et al., 2015) and other similar studies (Peters and Pereira, 2017). The high latent correlations are compatible with a theory of giftedness that regards it as variably manifested across domains with an underlying shared ability aspect, according to the authors of the Spanish adaptation (Rosado et al., 2015), who go into more detail about this topic. This idea would lead to a bi-factor model with six distinct elements, according to Benson and Kranzler (2018) and Jaburek et al. (2022). In addition, the results of our bi-factor model demonstrate that the GRS-S scales primarily reflect different aspects of giftedness in addition to general cognitive ability, which is consistent with the findings of Petscher and Pfeiffer (2019). This concept would result in a bi-factor model with six distinct factors, which is also supported by Benson and Kranzler (2018) and Jaburek et al. (2022). Parallel to this, our bi-factor model results show that the GRS-S scales predominantly reflect various dimensions of giftedness in addition to general cognitive ability, which is in line with Petscher and Pfeiffer (2019) findings. According to Peters and Pereira (2017) and Jaburek et al. (2022), the findings of the present study may suggest that the GRS-S reflects a general cognitive ability with multiple dimensions of giftedness. Furthermore, our findings are consistent with the GRS-P in Greek preschoolers, which demonstrated that the application of EFA and CFA revealed the presence of a sizable general factor and that it accounted for a sizable amount (82.95%) of the GRS-P variance (Sofologi et al., 2022). Additionally, our findings are consistent with Pfeiffer and Jarosewich (2003) findings according to which the value of the internal consistency reliability index was found to be excellent for all subscales, making the Greek GRS-S a reliable screening tool for measuring giftedness in elementary and middle school children.

Similarly, the findings of the Italian translated version confirmed the excellent internal consistency reliability index for all sub-scales (0.95–0.96) as well as a general factor with a multidimensional character confirmed with the exploratory analysis of factors (Sisto et al., 2022). The findings above were also supported by the Slovene translation of the GRS-S (Lep and Busik, 2020). The results of this study lend credence to the idea that teachers may be equally likely to consider a variety of student characteristics when rating them, including social–emotional skills, motor skills, and artistic abilities (Gresham et al., 2008; Schoemaker et al., 2008; Lep and Busik, 2020; Sisto et al., 2022). Furthermore, it is crucial to consider the psychometric properties of these factors even though a dimensional bi-factor model may best represent GRS-S scales (Benson and Kranzler, 2018; Sofologi et al., 2022).

There is a distinct difference between dimensionality and interpretability, according to Rodriguez et al. (2016) because the constituent elements of multidimensional scores must have sufficient identifiable, reliable variance to support interpretation. Because of this, one dimension may account for a significant percentage of the variance while the other may account for a much smaller portion. According to a study by Benson and Kranzler (2018), the general rating factor also explained 80% of the variance on the GRS-P and 72% of the variance on the GRS-S, suggesting that the vast majority of what the GRS measures is a general broad component that reflects a multidimensional profile. We further hypothesize that teachers use multidimensional ratings from different angles, ranging from cognitive capabilities or academic achievement to a more comprehensive belief of students’ profiles, based on characteristics like academic performance, behavior, and creativity (generally within the academic environment). Real academic proficiency and effectiveness consequently represent a child’s cognitive abilities (working memory, fluid intelligence, reasoning, learning efficiency, etc.), as well as other non-cognitive characteristics (motivation, social support, etc.), academic knowledge, and non-intellectual abilities (Jarosewich et al., 2002; Foley-Nicpon et al., 2011). Since both intellectual potential and non-academic abilities (creativity, academic passion, perseverance, and motivation) can be used to understand giftedness, we support the idea that the interpretations are consistent with the tripartite model of giftedness. Studies focused on the technical applicability of the GRS have been reported in the literature (Margulies and Floyd, 2004; Benson and Kranzler, 2018), while questions concerning the construct validity of the GRS are still being discussed.

Considering the fact that the findings of factor analyses of the GRS’s internal structure in the United States have been published high positive correlations are seen among all of the GRS scales (Benson and Kranzler, 2018; Petscher and Pfeiffer, 2019). Additionally, considerable correlations between several relevant external criteria and the GRS scales do not provide strong support for their validity (Sisto et al., 2022). It has been found that both GRS scales correlate well with a variety of intelligence measures that are attributed to a general component. Petscher and Pfeiffer (2019), in contrast, showed a six-factor structural model demonstrating a multidimensional character of giftedness in their condensed or abbreviated form of the GRS-S (30 items utilizing a 3-point rating scale) (Benson and Kranzler, 2018). The authors of the Czech GRS-S adaptation assert that their confirmatory model complies with the original structure and that the high latent correlations between the factors, particularly Intellectual Ability, Academic Ability, and Motivation, are a fact that mirrors the multidimensional nature of giftedness (Jaburek et al., 2022). However, these associations resemble those that both the original authors (Pfeiffer and Jarosewich, 2003) and later modifications reported (Pfeifer et al., 2004; Lee and Pfeiffer, 2006; Li et al., 2008; Rosado et al., 2015). The GRS-S reviewers have previously brought out this problem (Margulies and Floyd, 2004), and it is one of the reasons why other models have been put up and tested (Li et al., 2008; Rosado et al., 2015), all of which showed a good fit. Additionally, the analysis of the correlations between the GRS-S scales revealed that all six scales had a statistically significant positive association with one another.

The moderate/high positive correlations of the GRS-S discovered in current research confirm the findings of Pfeiffer and Jarosewich (2003) and Petscher and Pfeiffer (2019). According to Pfeiffer and Jarosewich (2003), despite showing distinct manifestations in the various realms of human performance, this pattern of interactions is consistent with the multifaceted nature of giftedness. The current research supports the hypothesis that evaluating non-intellectual parameters of giftedness (and/or intellectual parameters of giftedness which are beyond IQ tests), such as creativity, artistic talent, leadership, and motivation, is a key criterion for the precise identification of giftedness (Acar et al., 2016). These criteria reflect characteristics and skills that are most easily seen in classroom settings and in a child’s academic achievement, and as a result, are more likely to be noticed by teachers (Foley-Nicpon et al., 2011; Benson and Kranzler, 2018). The findings in this study provide evidence for the factorial validity of GRS-S and suggest that gifted students may be easier to spot in a Greek context. Additionally, our results for the uni-factorial and bi-factor models lend credence to the assumption that every GRS scale largely captures the same global competencies associated with giftedness (Margulies and Floyd, 2004). A substantial body of research from the past two decades shows the importance of evaluating academic aptitudes, creativity, and leadership to provide a thorough and reliable assessment of gifted students (Jarosewich et al., 2002; Pfeiffer, 2002; Kornmann et al., 2015; Pfeiffer, 2015; Machts et al., 2016). Teachers’ evaluations provide crucial information that must be considered when assessing whether a child fully satisfies the necessary gifted requirements. Furthermore, information from both conventional IQ tests and instructors’ best assumptions should be used to create an accurate gifted profile for each student. Multidimensional assessments based on a student’s complete academic career will eventually be accepted by the field of gifted identification research, claim Renzulli and Renzulli (2010) and Lim et al. (2010). Research has revealed that teachers’ perspectives are important for recognizing gifted children since they are involved in their student’s daily educational process. However, research has shown that since teachers are actively involved in their daily educational process, their opinions are vital for identifying gifted learners (Lim et al., 2010). The GRS-School Form provides the teacher with all the relevant information in the identification process and favors the connection between evaluation and later intervention offering many indicators about the characteristics of the programs and services that can best meet the needs of gifted children. It also supports the connection between evaluation and later intervention. Additionally, as teachers are involved in the identification process, naturally, this makes it easier for educators and psychologists to work together on creating effective gifted educational programs and services for gifted children.

One limitation of the current study is the fact that we did not apply a substantial reduction in the number of items from 72 to 30 and in the complexity of the rating scale, from a 9- to a 3-point scale (low, medium, and high) according to the findings of Petscher and Pfeiffer (2019). Specifically, Petscher and Pfeiffer (2019) stressed that a brief version of the GRS-S could be achieved, without sacrificing reliability or validity, with as few as 30 items using a 3-point rating scale. According to the researchers, a brief form of the GRS-S can be used as a universal or selective screener for giftedness without sacrificing key psychometric considerations. Future work may consider evaluating the extent to which GRS-S scales could be reduced without sacrificing the reliability of scores in the Greek context to verify (at item-level data) the GRS-S organization in the proposed by Pfeiffer and Jarosewich (2003) six-factor structure. Further, according to Benson and Kranzler (2018), the test of the internal structure of GRS-S would be more well-suited to exploratory structural equation modeling (ESEM) with target rotation (Asparouhov and Muthén, 2009; Marsh et al., 2009, 2014). Therefore, a limitation of the present study is the effect of the use of traditional confirmatory factor analyses (CFA) as the way to test the factor structure of the GRS-S. As a result, in a future study would be useful for the Greek version of GRS-S to be analyzed with the ESEM in the population of Greek elementary and middle school teachers. Regarding the rest study’s shortcomings and research limitations, we did not investigate how other factors, such as students’ academic achievement, children’s performance on psychometric exams, children’s behavior in classrooms, teachers’ gender, or years of schooling, might affect teachers’ assessments. The examination of each of these factors might support the conclusions we have already drawn. Because this exploratory study is part of a wider experimental design, more research is required to evaluate whether the Gifted Rating Scales are a useful diagnostic tool in the Greek educational context. Despite its limitations, this study is the first to discuss the GRS-S’s validation in the Greek setting. Our findings support the factorial validity of the GRS-S and can be applied to the evaluation of gifted students in the Greek community.

## 6. Conclusion

As a result, this study contends that by giving different sources of information on gifted students, instructors’ assessments can reinforce and improve the accuracy of the identification procedure. In this regard, teachers’ assessments of talented students are crucial because their exceptional abilities sometimes cannot be assessed using only conventional IQ testing (Jarosewich et al., 2002; Robertson et al., 2011; Erwin and Worrell, 2012; Slater, 2018). Based on our preliminary findings and the evaluation of gifted learners in the Greek cultural context, which should incorporate psychometrically sound measurements and information from teachers’ estimations, our research demonstrates the structural validity and the internal consistency reliability of the Greek version of the Gifted Rating Scales - School Form as a diagnostic instrument for gifted students. The GRS-S appears to be a useful brief screening tool to be taken into account in Greece’s gifted education programs because it is affordable and simple to use. Educators would welcome a screening tool that takes less time to complete, especially when used in classrooms to screen large numbers of students for full gifted testing, early school admissions decisions, and determining appropriate grade placement and or acceleration decisions. Since having access to scientific identification tools is a start in the right direction for gifted education in Greece, the study’s practical implications include the availability of a reliable and valid tool for identifying prospective giftedness.

## Data availability statement

The raw data supporting the conclusions of this article will be made available by the authors, without undue reservation.

## Ethics statement

The studies involving humans were approved by Scientific and Ethics Committee of the University of Ioannina (25847/01/06/2021). The studies were conducted in accordance with the local legislation and institutional requirements. The participants provided their written informed consent to participate in this study.

## Author contributions

MSo, AP, and GP: conceptualization. MSo and GP: methodology. MSo, DM, GP, and MSt: validation. TA, AD, TF, AL, A-RG, AT, DM, GP, and MSt: formal analysis. TA, AL, AT, and GP: investigation. MSo and GP: resources. MSo, HZ, GK, GN, KS, PV, and DM: data curation. MSo and GP: writing—original draft preparation. MSo, DM, and GP: writing—review and editing. MSo, TF, HZ, PV, KS, GK, AT, AL, A-RG, DM, and MSt: visualization. GP: supervision. MSo, TA, AD, AL, A-RG, GN, AP, and GP: project administration. All authors contributed to the article and approved the submitted version.
